# Preliminary Evidence for Training-Induced Changes of Morphology and Phantom Limb Pain

**DOI:** 10.3389/fnhum.2017.00319

**Published:** 2017-06-20

**Authors:** Sandra Preißler, Désirée Thielemann, Caroline Dietrich, Gunther O. Hofmann, Wolfgang H. R. Miltner, Thomas Weiss

**Affiliations:** ^1^Department of Biological and Clinical Psychology, Friedrich Schiller UniversityJena, Germany; ^2^Clinic for Trauma and Reconstructive Surgery, Berufsgenossenschaftliche Kliniken Bergmannstrost HalleHalle, Germany; ^3^Department of Trauma, Hand and Reconstructive Surgery, University Hospital JenaJena, Germany

**Keywords:** gray matter, plasticity, prosthesis, somatosensory feedback, upper limb amputation

## Abstract

The aim of this study was to investigate whether a special prosthetic training in phantom limb pain patients aimed at increasing the functional use of the prosthesis leads to neural morphological plasticity of brain structures and a reduction in phantom limb pain. For chronic pain disorders, it was shown that morphological alterations due to pain might become at least partially reversed by pain therapies. Phantom limb pain is a chronic pain disorder that is frequently followed by neural plasticity of anatomical brain structures. In our study, 10 patients with amputation of the upper limb participated in a two-week training with a myoelectric prosthesis with somatosensory feedback. Grip strength was fed back with electrocutaneous stimulus patterns applied to the stump. Phantom limb pain was assessed before and after the two-week training. Similarly, two T1 weighted MRI scans were conducted for longitudinal thickness analyses of cortical brain structures. As result of this treatment, patients experienced a reduction in phantom limb pain and a gain in prosthesis functionality. Furthermore, we found a change of cortical thickness in small brain areas in the visual stream and the post-central gyrus ipsilateral to the amputation indicating morphological alterations in brain areas involved in vision and pain processing.

## Introduction

After amputation of a limb, up to 80% of patients experience painful sensations in the missing body part (Desmond and Maclachlan, 2010). Such painful sensations are termed phantom limb pain (PLP; Mitchell, 1871).

Previous studies have indicated structural and functional alterations in different parts of the neuraxis following amputation (Flor et al., 2006). Functional plasticity was shown mainly in the primary somatosensory cortex (SI; Merzenich et al., 1984; Pons et al., 1991; Weiss et al., 1998, 2000; Lotze et al., 2001; Flor et al., 2006). It was assumed that sensory areas formerly representing the amputated structure of the body became “enslaved” by the neural representations of neighboring body structures in SI or still receive some input from the stump and other parts of the afferent neural system. The amount of such functional cortical reorganization was highly correlated with PLP intensity (Flor et al., 1995).

Besides such functional plasticity in SI, neural plasticity of other anatomical structures was identified following amputation (Draganski et al., 2006; Preißler et al., 2013b). Draganski et al. (2006) showed additional decrease in gray matter within the thalamus contralateral to the amputated side. Gaser et al. (2004) found decreased gray matter in primary motor cortex (MI), SI, superior parietal cortex contralateral to the amputated limb. Additional decrease in gray matter was found in the supplementary motor area (SMA), and the ipsilateral primary motor cortex and bilaterally in the cerebellum and the brain stem. Moreover, amputation in upper limb amputees is associated with a reduction of gray matter in areas in MI representing the amputated limb and with an increase of gray matter in parts of the cortex belonging to dorsal and ventral visual streams (Preißler et al., 2013b). Furthermore, patients with high PLP compared to patients with low PLP showed reduced gray matter in the right insular cortex, dorsolateral prefrontal cortex (DLPFC) and orbitofrontal cortex and increased gray matter in the caudal anterior cingulate cortex (ACC; Preißler et al., 2013b), cingulate cortex, insula, temporal lobe, and frontal/prefrontal cortex (May, 2011b). The cortical decrease was associated with the duration of chronic pain in most of these studies (Chan et al., 2004; Mendola et al., 2005; Draganski et al., 2006; Wrigley et al., 2009 for review see May, 2011b). Therefore, May (2011b) suggests that the morphological changes are at least partly a consequence of constant pain in chronic pain disorders.

Despite a large quantity of approaches exist to handle PLP, PLP is still difficult to treat (Sherman, 1997; Subedi and Grossberg, 2011). Common treatments are pharmacological approaches, physical therapy or other concepts including biofeedback, hypnosis, and chiropractic care. However, several patients reported an increase of PLP and/or stronger impairment of mood in everyday life after these interventions (Hanley et al., 2006).

One treatment whose rational was derived from research of the neural mechanism of PLP is the sensory discrimination training (Flor et al., 2001). It affects the functional reorganization processes in SI following amputation (Flor et al., 2001; Flor, 2002). Flor et al. (2001) studied patients with upper limb amputation. The patients learned to discriminate the position and frequency of electrocutaneous stimuli transmitted via eight electrodes onto their stump. The trained patients showed a gain in discrimination ability, which was negatively associated with cortical reorganization of the lip (which is the neighboring area with respect to the arm) and PLP intensity.

Besides this specific treatment, there is evidence that the usage of a prosthesis could influence PLP in a positive manner. Weiss et al. (1999) showed that wearing a functional Sauerbruch prosthesis is associated with a larger amount of functionality and a reduced intensity of PLP compared to a cosmetic prosthesis. In addition, Lotze et al. (1999) showed that the usage of a myoelectric prosthesis seemed to have an affirmative impact on the intensity of PLP as well. The association between usage of a prosthesis and PLP seems to be mediated by functional cortical reorganization (Lotze et al., 1999). Thereby, increased use of a prosthesis may be accompanied by decreased PLP.

Recent studies demonstrated that intensive behavioral training significantly affects the morphology of brain areas (Draganski and May, 2008; May, 2011a). For example, Draganski et al. (2004) trained healthy participants for 3 months in juggling. After the training, they observed a gray matter increase in the extrastriate motion specific area bilaterally and in the left inferior parietal sulcus of the jugglers.

In addition to the effects of training on structural plasticity, structural alterations following pain resolving therapies are discussed (Obermann et al., 2009; Rodriguez-Raecke et al., 2009; Seminowicz et al., 2011). Rodriguez-Raecke et al. (2009) demonstrated gray matter decreases in several brain regions, e.g., amygdala and brainstem, in patients with hip osteoarthritis compared to healthy controls. Patients successfully undergoing pain surgery showed a gray matter increase of the formerly decreased brain regions. Such partial reversal of gray matter after pain relief has been demonstrated also for patients with low back pain (Seminowicz et al., 2011) and for patients with chronic post-traumatic headache (Obermann et al., 2009).

A therapeutic training, which includes the above-described sensory discrimination training and a training with a prosthesis, may potentially influence both PLP and anatomical cortical reorganization processes. One possible training combines the myoelectric prosthesis with somatosensory feedback (SF) introduced by Dietrich et al. (2012). They examined eight upper limb amputees participating in a two-week training with a myoelectric hand prosthesis providing SF on grip strength. All patients showed a significant increase of functionality and a significant reduction of PLP.

Based on these studies, the aim of the present study was two-fold: First, we examined whether a two-week training with a myoelectric prosthesis with SF is followed by a reduction of PLP and a gain in functionality. Second, we investigated whether these effects might be associated with structural alterations of the cortical thickness of different brain areas in patients who successfully participated in our training.

## Materials and methods

### Participants

Ten patients (one female) with right forearm amputation participated in this study. Exclusion criteria were plexus avulsion, amputation of another part of the body, congenital malformation, and/or history of a neurological or psychiatric disease. The patients were on average 43 years old. Further, sociodemographic and clinical data of patients are presented in Table 1. Amputation was undertaken following a traumatic accident in all patients on average 8.53 years before the present study. All patients were used to a standard myoelectric prosthetic system before inclusion in the study. All procedures were conducted in accordance with the ethical standards of the 1964 Helsinki declaration and its later amendments or comparable ethical standards. The study was approved by the Ethics committee of the Friedrich Schiller University. Written informed consent was obtained from each subject prior to participation.

**Table 1:** Sociodemographic and clinical data of patients.

**Gender**	**Side of amputation**	**Age in years**	**Time since amputation in months (b.a.)**	**Time of prosthesis use in months (b.a.)**	**PLP rating (VAS_pre_ in %)**
M^D^	R	39	84	84	28.00
M^D^	R	28	18	9	6.65
M^D^	R	43	188	183	50.96
M	R	24	44	27	0.00^*^
M	R	38	58	54	30.49
M	R	57	337	335	3.91
M	R	34	110	98	2.09
M	R	59	14	9	65.97
M	R	57	64	36	20.60
F	R	51	107	105	63.92

b.a., before assessment; PLP, phantom limb pain; VAS_pre_, visual analog scale mean rating on the day before the training; M, male; F, female; R, right; L, left;

* *patient reported occasional PLP, but no PLP at the time of examination; ^D^indicates that patients where also included in the study of Dietrich et al. (2012)*.

### Assessment of phantom limb pain

Assessment of PLP was performed using a 10 cm visual analog scale (VAS) with “no pain at all” presented by the left end point and “the worst pain I can imagine” by the right end point of the scale (Scott and Huskisson, 1976). PLP often occurs intermittently and has daily as well as situational variability. Therefore, PLP was rated at different time points per day using two different conditions (patient wore the prosthesis and when the prosthesis was not been worn by the patient). More specifically, the VAS assessment was conducted in the following way: In the morning, there were four ratings: (1) before the first assessment with the prosthesis, (2) before the first assessment without the prosthesis, (3) after this assessment without the prosthesis, (4) after this assessment with the prosthesis. At noon, there was one assessment with the prosthesis. In the afternoon, there were again four ratings: (1) before the MRI assessment with the prosthesis, (2) before the MRI assessment without the prosthesis, (3) after the MRI assessment without the prosthesis, (4) after the MRI assessment with the prosthesis. Based on these nine values, a mean pain value was conducted for the day just before the training (VAS_pre_) and on the day just after the training (VAS_post_).

As a second measure of PLP, patients were requested at the end of the training to estimate the change of pain intensity (CPLP) experienced since the start of intervention using a two-pole 10 cm scale with “strongly reduced” and “strongly increased” at the endpoints of the scale and “no change” in the middle of the scale.

### Prosthetic training

Patients took part in a daily prosthesis training for 10 consecutive working days. Each training session lasted 3.5 h, with an additional 20 min break. The prosthesis was equipped with SF. A detailed description of the training and the myoelectric prosthesis with SF is given in Dietrich et al. (2012). Shortly, the prosthesis allows a measure of grip strength through pressure sensors located in the bend between thumb and index finger of the prosthetic hand. Subject's grip strength is transformed into electric signals through the stimulation component of the device. This component has outputs for eight electrodes. The electrodes are applied at the patient's stump. They deliver electrocutaneous stimulus patterns to the skin of patient's stump for SF of the grip strength. Each electrocutaneous stimulus pattern is associated with predefined grip strength of the myoelectric prosthetic system. Electrocutaneous feedback is applied accordingly to our previous reports (Weiss et al., 2007; Walter-Walsh et al., 2009).

### Assessment of training success

To evaluate the effects of the sensory feedback myoelectric prosthesis, motor improvement was operationalized using two standardized tests based on the standard test procedure of constraint-induced movement therapy (CIMT; Bauder et al., 2001). The first task was a “pin board task” (T_PB; D'alessio and Spence, 1963) with the board located in front of the patient in a standardized position. The pins were positioned right above the board. The patient was requested to put as many pins as possible into the pin board within 1 min. The second task was a modification of the “tower of Hanoi” task (T_TH; Lucas, 1889). The patient was asked to move as many wooden discs as possible from the leftist to the rightist bar within 30 s and in a succeeding task from the rightist bar to the leftist bar, again within 30 s. Both the T_PB and T_TH tests were performed on the first (T_xx_begin_) and on the last training day (T_xx_end_).

Furthermore, patients were requested to provide ratings on a 5 point Likert scale ranging from 1 (“appropriate”) to 5 (“not at all”) at the first and at the last day of training in response to the following statements:

“I can interpret and evaluate the electrocutaneous feedback very well” (Q_EF); “I succeeded in muscular triggering of the myoelectric prosthesis” (Q_MT); “I feel confident about using the myoelectric prosthesis” (Q_CO).

### Behavioral data analysis

To evaluate the modification of PLP, we conducted the difference (ΔVAS) between VAS_pre_ and VAS_post_. We screened ΔVAS and CPLP for normal distribution using the Shapiro-Wilk test. We examined ΔVAS and CPLP with a one-sample *t*-test when the assumption of normality holds or with the Wilcoxon rank sum test when normality failed. For the assessment of training success, analyses of variance for repeated measurements were used with T_PB, T_TH, Q_EF, Q_MT, and Q_CO as dependent variables. The significance levels were set to *p* = 0.05 (two tailed). All analyses were conducted with IBM SPSS Statistics 23 (SPSS Inc., an IBM Company, Chicago, IL, USA).

### MRI data acquisition

Two T1-weighted sagittal oriented sequences for morphometric analyses were acquired for all subjects (192 slices; flip angle: 30°; matrix: 256 × 256; voxel size: 1 × 1 × 1 mm) before and after the prosthetic training. As subjects were included since 2005, the first four subjects were measured on a 1.5-T MRI scanner (Siemens Magnetom Vision Plus, Erlangen, Germany; TE: 5 ms; TR: 15 ms). For the remaining six participants, a 3-T MRI scanner (Siemens Magnetom Trio, Erlangen, Germany; TE: 3.03 ms; TR: 2.3 ms) was used. To reduce a possible field strength bias we controlled our participants' membership to the two different scanner types (Han et al., 2006). The patients are well-balanced to the two different field strengths (Table 2). Nevertheless, the same MRI scanner was used for the images of each subject before and after the training. All acquisitions were performed with a standard head coil to acquire whole brain MRI data.

**Table 2:** Balancing to the different scanner types.

	**Vision**	**Trio**	**Group difference test**
**GENERAL PARAMETERS**			
N male/female	4/0	5/1	χ^2^(1) = 0.74 (*p* = 0.39)
Age in years, M (*SD*)	33.3 (8.7)	49.5 (10.5)	*z* = 1.93 (*p* = 0.07)
Handedness (before amputation) right/left	4/0	6/0	–
**GENERAL CLINICAL PARAMETERS**			
BDI M (*SD*)	9.0 (12.9)	8.3 (5.7)	*z* = 0.74 (*p* = 0.56)
VAS_pre_, M (*SD*)	17.8 (18.2)	31.3 (26.1)	*z* = 0.43 (*p* = 0.76)
**AMPUTATION SPECIFIC PARAMETERS**			
Time since amputation in month, M (*SD*)	66.0 (78.8)	120.7 (116.7)	*z* = 1.07 (*p* = 0.35)
Mean age at amputation in years, M (*SD*)	27.5 (4.5)	39.5 (13.7)	*z* = 1.39 (*p* = 0.17)

*M, mean; SD, standard deviation; VAS_pre_, visual analog scale mean rating on the day before the training; BDI, sum score of Becks Depression Inventory (Beck et al., 1996)*.

### Cortical reconstruction and thickness segmentation

Cortical reconstruction and thickness segmentation were performed with the FreeSurfer image analysis suite (FreeSurfer 5.1, http://surfer.nmr.mgh.harvard.edu). To extract reliable volume and thickness estimates, images were automatically processed with the longitudinal stream in FreeSurfer (Reuter et al., 2012). The process includes three different steps: Firstly, all scans of different time points are processed independently in a cross-sectional analysis. Secondly, a base image of the person's brain anatomy is created. This is done by using all cross-sectional scans to create an unbiased within-subject template space and image using robust, inverse consistent registration (Reuter et al., 2010). More specifically, a median image is conducted which functions as an average image across all time points. Using this average image, all subsequent segmentations can be done on the approximated subject's anatomy of the brain. Thirdly, the longitudinal analyses take place where each time point is processed with respect to the within-subject template. This means, the difference to the template is observed and analyzed. The use of such a matching between average images and individual time point provides the advantage to automatically correct for parameters, which are the same in all time points for the subject. Several processing steps, such as skull stripping, Talairach transforms, atlas registration as well as spherical surface maps and parcellations are then initialized with common information from the within-subject template, significantly increasing reliability and statistical power (Reuter et al., 2012). Continuative statistical analysis was based on the resulting difference variable and done with mri_glmfit.

### Longitudinal analysis of thickness data

To test region-specific structural gray matter changes between the two time points, we modeled regressions within the mri_glmfit tool using the general linear model. Intercepts and slopes of the regression were estimated. Regions that showed a significant difference at a voxel-level of *p* < 0.05 between the two time points, were streamed out and mapped into Talairach space. We balanced between the opportunities of type I (α) error and type II (β) error using the recommendations of Lieberman and Cunningham (2009), i.e., a combination of intensity (*p* < 0.005) and spatial extend (>10 vertices). Regression coefficients of significant clusters were streamed out with their individual *F*-value for testing the estimator against zero.

Firstly, we used a regression to test the main effect of the prosthetic training (Equation 1).

(1) $$\left(\begin{array}{ccc} {\text{y}}_{11} & \cdots & {\text{y}}_{\text{1v}}\\ \vdots & \ddots & \vdots \\ {\text{y}}_{n1} & \cdots & {\text{y}}_{n\text{v}} \end{array}\right)=\left(\begin{array}{c} \begin{array}{c} 1\\ 1 \end{array}\\ \begin{array}{c} \vdots \\ 1 \end{array} \end{array}\right)\left(\beta_{0}\right)+\left(\begin{array}{ccc} \varepsilon_{11} & \cdots & \varepsilon_{\text{1v}}\\ \vdots & \ddots & \vdots \\ \varepsilon_{n1} & \cdots & \varepsilon_{n\text{v}} \end{array}\right)$$

Equation (1) shows regression for examining the main effect. *n* represents the patients and *v* represents the vertices.

Secondly, we examined the influence of ΔVAS on the effect of training between two measurement periods with the second regression (Equation 2).

(2) $$\left(\begin{array}{ccc} {\text{y}}_{11} & \cdots & {\text{y}}_{\text{1v}}\\ \vdots & \ddots & \vdots \\ {\text{y}}_{n1} & \cdots & {\text{y}}_{n\text{v}} \end{array}\right)=\left(\begin{array}{cc} \begin{array}{c} \begin{array}{c} 1\\ 1 \end{array}\\ \begin{array}{c} \vdots \\ 1 \end{array} \end{array} & \begin{array}{c} \begin{array}{c} \Delta {\text{VAS}}_{1}\\ \Delta {\text{VAS}}_{2} \end{array}\\ \begin{array}{c} \vdots \\ \Delta {\text{VAS}}_{n} \end{array} \end{array} \end{array}\right)\left(\begin{array}{c} \beta_{0}\\ \beta_{1} \end{array}\right)+\left(\begin{array}{ccc} \varepsilon_{11} & \cdots & \varepsilon_{\text{1v}}\\ \vdots & \ddots & \vdots \\ \varepsilon_{n1} & \cdots & \varepsilon_{nv} \end{array}\right)$$

Equation (2) shows regression for examining the effect of PLP change. *n* represents the patients and *v* represents the vertices.

This covariate analysis allows two different subanalyses. First, we investigated the main effect of the prosthetic training adjusted for PLP change (β_0_). Second, we investigated the regression coefficient due to the interaction between structural changes within the training and PLP change (β_1_) for estimating the effect of PLP change on the morphometry besides the training effect. The resulting regression coefficient β_1_ can be interpreted as follows: A negative coefficient regarding a reduction in gray matter indicates that decreased subjective pain ratings were associated with more reduction of gray matter. A positive coefficient indicates that decreased subjective pain is associated with less reduction in gray matter. A negative coefficient regarding an increase of gray matter means that decreased subjective PLP is associated with more increase in gray matter. A positive coefficient shows that decreased subjective pain ratings are associated with less increase in gray matter. In this analysis reduction and increase on cortical thickness are not two extremes of one dimension. They are two different predicted dependent variables. However, reduction and increase of subjective PLP are two extremes of the dimension difference in pain (post-prae).

## Results

### Behavioral data

#### Phantom limb pain

We found a significant reduction of PLP following the training with both pain measures (VAS: Mean_pre_ = 2.73, *SD*_pre_ = 2.54, Mean_post_ = 2.30, *SD*_post_ = 2.60; *z* = −2.07, *p* < 0.05; CPLP: Mean = −2.64, *SD* = 2.70; *t*_df = 9_ = −3.8, *p* < 0.05).

#### Training outcome measures

After the prosthetic training, patients showed a gain in the pin board task (Mean_pre_ = 6.4, *SD*_pre_ = 4.3, Mean_post_ = 11.7, *SD*_post_ = 5.8; Λ_df = 1.11_ = 12.6, *p* < 0.05) and the Tower of Hanoi task (Mean_pre_ = 6.1, *SD*_pre_ = 4.4, Mean_post_ = 14.4, *SD*_post_ = 7.0; Λ_df = 1.11_ = 29.4, *p* < 0.05).

Patients also showed improved performance according to the statements: “I can interpret and evaluate the electrocutaneous feedback very well”: Mean_pre_ = 6.0, *SD*_pre_ = 1.8, Mean_post_ = 8.5, *SD*_post_ = 1.4; Λ_df = 1.13_ = 0.36, *p* < 0.05; “I succeeded in muscular triggering of the myoelectric prosthesis”: Mean_pre_ = 7.1, *SD*_pre_ = 1.5, Mean_post_ = 8.7, *SD*_post_ = 1.4; Λ_df = 1.13_ = 0.63, *p* < 0.05; “I feel confident about using the myoelectric prosthesis”: Mean_pre_ = 7.0, *SD*_pre_ = 1.4, Mean_post_ = 8.8, *SD*_post_ = 1.1; Λ_df = 1.13_ = 0.38, *p* < 0.05.

### Morphometric analyses

#### Longitudinal analysis

**Figure 1:**
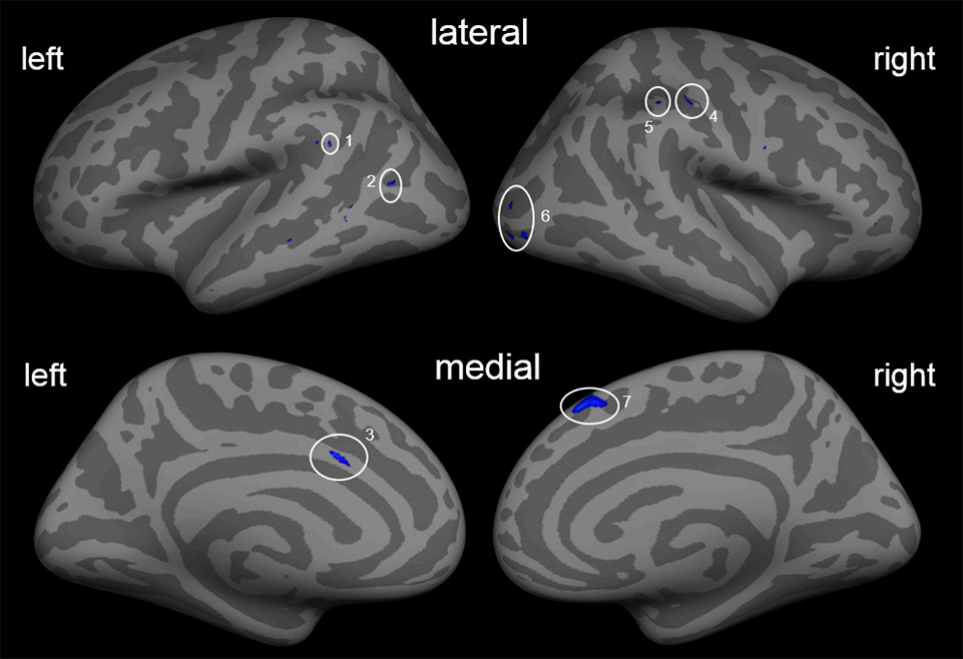
Cortical thickness differences due to the training. Inflated presentation of a standardized brain. Areas shown in blue are regions with decreased cortical thickness after the training. Only areas with a vertex-wise threshold of *p* > 0.05 are shown. 1, supramarginal; 2, middle temporal cortex; 3, anterior cingulate cortex; 4, post-central sulcus; 5, inferior parietal cortex; 6, occipital cortex; 7, superior frontal cortex.

Investigation of morphometric changes due to the prosthetic training revealed cortical thickness reductions. We found a reduction of cortical thickness in the post-central sulcus ipsilateral to the amputation side, i.e., ipsilateral to the trained arm. In addition, there is also a reduction of cortical thickness in the left middle temporal cortex, left ACC, right occipital cortex, and right superior frontal cortex (Figure 1; Table 3).

**Table 3:** Main effect of training.

**Region**	**Size (mm^2^)**	**Maximal activation (*z*-value)**	**β_0_**	***F*_*df* = 9_**	**Talairach coordinates**		
					***x***	***y***	***z***
**LEFT HEMISPHERE**							
Anterior cingulate cortex	21.83	−3.979	−0.1098	15.1902	−6.1	14.5	28.7
Superior frontal cortex (BA 9)	5.72	−2.613	−0.1713	12.0118	−16.0	43.5	33.0
Middle temporal cortex	7.51	−2.82	−0.0801	11.8042	−42.0	−62.8	12.9
	6.17	−2.751	−0.1257	13.0109	−59.0	−46.6	25.9
**RIGHT HEMISPHERE**							
Superior frontal cortex (BA 8)	114.44	−3.861	−0.1375	19.2569	7.0	29.3	47.5
Post-central sulcus	13.04	−3.106	−0.0766	10.464	46.3	−20.5	38.0
Inferior parietal cortex (BA 40)	11.67	−2.895	−0.0972	13.8605	44.1	−32.2	40.3
Occipital cortex	9.19	−2.567	−0.1076	12.9698	28.8	−82.4	6.1
	27.74	−3.121	−0.1129	14.2215	30.9	−90.1	−3.9
	25.47	−3.358	−0.1066	15.1947	37.5	−83.1	−1.8

#### Influence of additional variables

To ensure that the results mentioned above are not caused by gender or age of the participants, we performed additional analyses. The results of these additional analyses are reported in the Supplementary Material (Figures S1, S2, Tables S1, S2). Only results, which are consistent, will be discussed.

#### Covariate analysis of longitudinal data

In order to evaluate morphometrical effects more detailed, ΔVAS was used as a covariate in a regression model (Equation 2). Accounting for ΔVAS, the results of cortical changes referring the prosthetic training allow two subresults.

**Figure 2:**
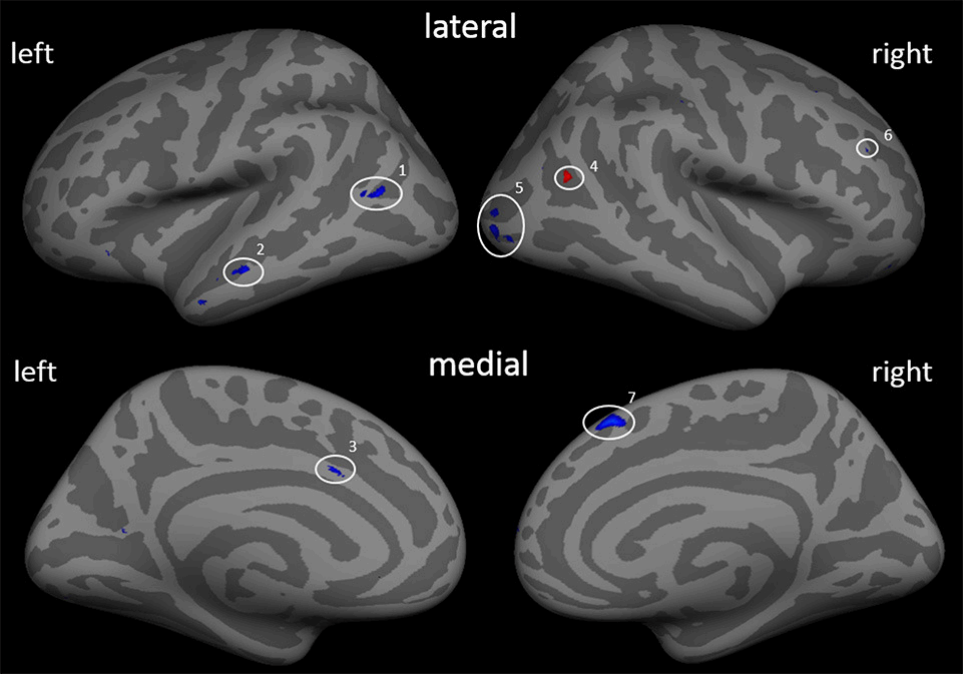
Cortical thickness differences due to the training adjusted by the covariate ΔVAS (β_0_). Inflated presentation of a standardized brain. Areas shown in blue are regions with decrease in thickness after the training. Red areas mark an increase in thickness. Only areas with a vertex-wise threshold of *p* > 0.05 are shown. 1 and 2, middle temporal cortex; 3, anterior cingulate cortex; 4, middle temporal cortex; 5, occipital cortex; 6, dorsolateral prefrontal cortex; 7, superior frontal cortex.

First, the effect of the training adjusted for PLP change can be specified (Figure 2; Table 4). After controlling for ΔVAS, a quite similar picture of morphological changes emerged compared to the results without controlling for ΔVAS. Besides the reductions in cortical thickness in the left middle temporal cortex, left ACC, right occipital cortex, and right superior frontal cortex, two additional areas showed changes in cortical thickness with a gain in cortical thickness in the right inferior parietal cortex (IPC) and a reduction of cortical thickness in the right DLPFC.

**Table 4:** Main effect of training adjusted for ΔVAS.

**Region**	**Size (mm^2^)**	**Maximal activation (*z*-value)**	**β_0_**	***F*_*df* = 9_**	**Talairach coordinates**		
					***x***	***y***	***z***
**LEFT HEMISPHERE**							
Anterior cingulate cortex	6.07	−3.114	−0.106	14.191	−6.1	14.5	28.7
Middle temporal cortex	7.99	−3.776	−0.079	14.712	−53.5	−0.3	−24.9
	13.00	−3.177	−0.106	14.186	−42.3	−62.2	12.9
	17.38	−3.069	−0.117	14.897	−56.7	−19.5	−12.4
**RIGHT HEMISPHERE**							
Superior frontal cortex (BA 8)	72.07	−3.597	−0.168	21.094	6.9	26.4	48.8
Dorsolateral prefrontal cortex	10.47	−3.385	−0.084	12.740	14.1	61.0	7.9
Inferior parietal cortex (BA39)	11.00	2.933	0.130	16.125	39.9	−56.1	20.3
Occipital cortex	34.12	−3.159	−0.121	13.754	29.9	−88.3	2.0
	7.33	−2.453	−0.142	13.598	28.4	−83.5	5.9

**Figure 3:**
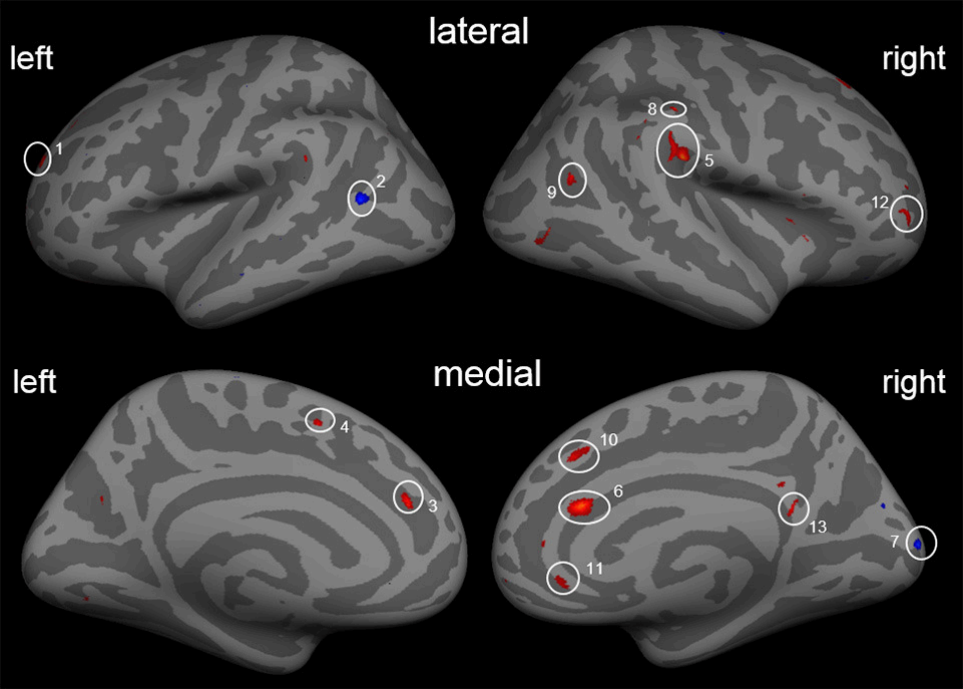
Cortical thickness differences due to the training with the covariate ΔVAS. Inflated presentation of a standardized brain. Areas shown in blue are regions with decrease in cortical thickness after the training. Red areas mark an increase in cortical thickness. Only areas with a vertex-wise threshold of *p* > 0.05 are shown. 1, superior frontal; 2, middle temporal cortex; 3, anterior cingulate cortex; 4, inferior frontal; 5, SII/Insula; 6, anterior cingulate cortex; 7, occipital cortex; 8, post-central sulcus; 9, middle temporal cortex; 10, superior frontal cortex; 11 and 12, dorsolateral prefrontal cortex; 13, posterior cingulate cortex.

Second, besides the gain of cortical thickness in the post-central sulcus (right hemisphere) as a function of ΔVAS, we found a gain in cortical thickness in areas of the ACC, the insula, and visual streams that were associated with changes in pain ratings. Additional reductions in the left middle temporal cortex and in the right occipital cortex were found (Figure 3; Table 5).

**Table 5:** Effects of covariate ΔVAS.

**Region**	**Size (mm^2^)**	**Maximal activation (*z*-value)**	**β_1_**	***F*_*df* = 8_**	**Talairach coordinates**		
					***x***	***y***	***z***
**LEFT HEMISPHERE**							
Anterior cingulate cortex	13.71	3.167	0.023	17.105	−13.3	36.0	17.4
Dorsolateral prefrontal cortex	58.49	3.416	0.014	18.479	−19.3	52.4	18.3
Middle temporal cortex	28.96	−3.403	−0.022	17.523	−47.3	−60.2	13.3
Inferior frontal gyrus (BA 11)	19.83	2.841	0.019	15.264	−16.9	35.3	−18.4
**RIGHT HEMISPHERE**							
SII/Insula	82.06	4.003	0.022	18.990	49.6	−25.2	25.8
Anterior cingulate cortex	48.15	3.932	0.027	19.966	6.8	26.2	17.8
Occipital cortex	46.95	−3.725	−0.022	21.814	9.9	−92.4	13.6
Post-central cortex	5.49	3.517	0.016	15.988	50.1	−27.1	38.2
Inferior parietal cortex (BA 39)	9.43	2.920	0.0199	16.183	39.9	−56.1	20.3
Superior frontal cortex (BA 8)	21.53	2.901	0.029	15.921	18.2	30.6	46.8
Dorsolateral prefrontal cortex	20.14	2.809	0.029	14.595	37.1	50.5	3.4
	11.24	2.729	0.020	13.652	8.4	28.1	33.8
Posterior cingulate cortex	7.38	2.784	0.016	12.550	5.7	−47.3	20.0

## Discussion

This study aimed at examinining the effects of a two-week prosthesis training of subjects with chronic PLP with a myoelectric SF prosthesis on PLP and on its underlying brain morphometry. On the behavioral level, subjects reported significantly reduced PLP, and a gain in functionality of prosthesis use. Morphometrically, we found small reductions of cortical thickness of the post-central gyrus on the hemisphere ipsilateral to the trained extremity and in areas of the visual streams. Effects on PLP were quite similar but not identical.

### Behavioral data

All patients reported significantly less PLP after the training. This goes in line with a former study by Dietrich et al. (2012) showing that “active” functional hand prostheses with additional SF significantly affect PLP. In addition, our results are in line with previous studies showing that the amount of prosthesis use is positively associated with reduction of PLP (Lotze et al., 1999; Weiss et al., 1999; Preißler et al., 2013a). Weiss et al. (1999) investigated nine patients using a Sauerbruch prosthesis and 12 persons wearing cosmetic prostheses indicating that usage of an “active” Sauerbruch prosthesis is accompanied by a reduction of PLP, whereas the use of the cosmetic prostheses had no impact on PLP. These results indicate that patients who use their residual limb with the prosthesis more often show less PLP. The results were supported by a study of Lotze et al. (1999). They showed that prosthesis use mediates the relationship between PLP and functional cortical reorganization. Patients who were not provided with a functional myoelectric prosthesis showed stronger PLP and a significant larger extent of functional cortical reorganization than patients using a myoelectric prosthesis.

Furthermore, the reduction of PLP supports results of Flor et al. (2001). Their patients who participated in a discrimination training showed a gain in discrimination ability, which was negatively associated with cortical reorganization and PLP (Flor et al., 2001). As our patients also received somatosensory discrimination in form of SF, somatosensory stimulation of the stump might have helped to reduce PLP additionally to the pure use of the myoelectric prosthesis.

An alternative interpretation for the observed PLP reduction after training also suggests an alteration in the body scheme of the patients caused by the training. It has been hypothesized that an incongruence of conscious motor intention, motor efferents, proprioception, and visual perception of the body might lead to PLP (Harris, 1999). Specifically, it was hypothesized that the divergence of motor efferents to the muscles and the lack of perceived visual motion of the missing body part could result in a mismatch in higher level processing structures that might be associated with pain (Harris, 1999; Reilly et al., 2006). The additional feedback from the myoelectric prosthesis might also have influenced the incongruence of conscious motor intention, motor efferents, proprioception, and visual perception of the body in a positive manner. Since the SF provides information about the movements of the myoelectric prosthetic hand, it also provides feedback about the motor intention. Ehrsson et al. (2008) postulate that an amputee would perceive the prosthesis as belonging to his own body when you stimulate the prosthesis and the stump simultaneously. The authors additionally point to the role of the former receptive fields of the hand in SI. The stimulation of the stump gives feedback in areas formerly presenting the hand. This enables an illusion. Wearing a myoelectric prosthesis, which gives SF on the stump, also provides feedback to areas formerly presenting the hand and therefore enables this illusion. Thus, SF might influence the impaired body scheme of an amputee in a positive manner leading to a reduction of PLP.

In addition to the reduction of PLP, a gain in functionality in all standard tests occurred. Clearly, our patients improved the handling of the myoelectric prosthesis and recognized the value of the SF. Probably, this gain of functionality might have helped to reduce PLP. By the training, patients were focused on the functionality and the function of the artificial hand itself. Therefore, their attention might have shifted from the pain toward the function of the prosthesis. Additionally, all patients mentioned the personal importance of the gain in functionality in a short interview after the training.

Taken together, the observed reduction of PLP might strongly relate to the active prosthetic use (Lotze et al., 1999; Weiss et al., 1999), the somatosensory discrimination training (Flor et al., 2001; Moseley et al., 2008) and/or to an altered body scheme of the patients (Harris, 1999). Probably, all three mechanisms contribute to the observed decrease in PLP experience.

### Morphological data

#### Cortical changes with regard to the training effect

Focusing on morphological changes following the prosthesis training, there are only a few changes in cortical thickness. Analysis revealed a decrease of cortical thickness in the hand area of the post-central sulcus of the right hemisphere (i.e., ipsilateral to the amputation). This might indicate a decreased use of the non-affected arm. This interpretation is in line with results observed with stroke patients (Liepert et al., 1998) following CIMT of the affected upper extremity, but also on the not-affected side. Liepert et al. (1998) demonstrated a reduction of the cortical motor output area elicited by TMS for the non-affected adductor brevis muscle. This was interpreted as evidence that the increased use of the stroke-affected extremity during training relieved the use of the non-affected extremity with the result that its cortical representation became smaller. Obviously, the training induced excessive use of the prosthesis carrying extremity might have led to a similar change in our amputees, i.e., a reduced cortical representation of the healthy, non-amputated extremity on the contralateral hemisphere due to the fact that the majority of everyday motor tasks formerly were mastered compensatory with the non-amputated extremity but became mastered during and following training primarily with the prosthesis carrying extremity. An alternative explanation could be seen in the results demonstrated by Grothe et al. (2017). These authors could show that an intensive unilateral upper limb motor training resulted in an improved performance of the trained arm. Additionally, they showed an improved performance of the untrained side. For both sides, they showed changes in the intracortical facilitation of M1. This decrease in intracortical facilitation of M1 was associated with a transfer of training-induced improvement of performance from the trained to the untrained hand (Grothe et al., 2017). Therefore, our results in the post-central gyrus ipsilateral to the amputation might alternatively result from a transfer of training-induced improvements for both sides and not from a loss in function of the not-affected and untrained side.

Moreover, we found reductions of cortical thickness in the temporal and the occipital cortex. These reductions of cortical thickness might be associated with an adaption of the visual system to the usage of the SF prosthesis. Before treatment, the amputees were forced to visually control movements and actions with their prosthesis compared to the use of the hand before amputation (Preißler et al., 2013a,b). The SF system of our prosthesis provides in addition to visual control SF so that patients need less visual control during grasping and other motor acts with their prosthesis. This reduced effort of visual control might have resulted in a decreased engagement of subjects' visual systems with the consequence that its neural structures became functionally decreased and morphometrically smaller.

We also found a decrease of cortical thickness in the mesial part of the superior frontal cortex (BA 8). This region is known to be involved in the prediction of uncertainty (Volz et al., 2003), i.e., of events in the world, which are beyond personal control. Volz et al. (2003) could demonstrate that this region is especially activated under uncertainty compared to certain predictions. Volz et al. (2003) hypothesized that the mesial superior frontal cortex modulates stimulus-response associations by error evaluation. For upper limb amputees, who use a prosthesis without SF, more errors arise during grasping and other motor acts with the prosthesis in natural settings. Without SF from the hand, the produced grip force by the myoelectric prosthesis is more or less unpredictable (Atkins et al., 1996). The unpredictable grip force might be associated with a stronger involvement and higher activation of the mesial BA 8. During our training, the myoelectric device with SF allows more certain predictions of grip force and other motor acts. Therefore, the involvement of the mesial BA 8 and its cortical thickness might have become reduced.

After the training, patients showed a reduction of cortical thickness in the ACC. The ACC is involved in very early orienting responses to noxious stimuli (Vogt et al., 2003) and important for orienting in space and spatial behaviors (Vogt et al., 2003). As the patients learn to use SF during training, the requirement of spatial control of operations with the prosthesis becomes reduced, resulting in a reduction of cortical thickness.

Many of these observations seem to contradict a number of earlier findings on the cortical effects of training in animals and humans (Draganski and May, 2008; Anderson, 2011). For example Draganski et al. (2004) showed that a daily juggling training of 3 months increased the gray matter volume of visuo-motor control areas of the brain. In our study, patients had only 2 weeks of training. While some studies indicated a morphometrical increase of the visual cortex (Kwok et al., 2011) others failed very likely to the fact the type of interventions vary significantly between studies (Lovden et al., 2013). Lovden et al. (2013), providing a review on morphological changes due to training, mentioned that the fastest increase in gray matter, using appropriate designs and appropriate analyses, has been detected after 8 weeks of training in the right insula. Therefore, our training period might have been too short to induce a training-related increase of brain structures.

An additional explanation for the observed cortical reduction could be that we investigated the effects of training in amputees and of changes in PLP in a single analysis. Both training and changes in chronic pain are known to provoke structural changes (Gaser et al., 2004; May, 2011b). On the one hand, amputation might be accompanied with a loss of cortical volume in different regions (Draganski et al., 2006; Preißler et al., 2013b). On the other hand, several studies showed decreases in gray matter in pain processing areas in patients suffering from chronic pain (May, 2011b). The next two sections will discuss the specific changes evoked by each of the two processes using the results of the covariate analyses.

#### Cortical changes with regard to the training effects controlling for PLP

Taking into account the PLP change from prior to post-training as a covariate (ΔVAS), the results are similar to the results of our former analysis: We found reductions in cortical thickness in the left middle temporal cortex, left ACC, right occipital cortex, and right superior frontal cortex. These results have been discussed above. As these results remain constant, we can conclude that these cortical changes are related to the training even when controlling for PLP change. Importantly, two other areas show changes in cortical thickness associated with the training when controlling for PLP change. We found a gain in cortical thickness in the right IPC and a reduction of cortical thickness in parts of the right DLPFC. The effects seem to be concealed by PLP change.

The right IPC of the visual paths is the only region showing an increase in cortical thickness. This region is discussed as being involved in regulating visual attention, especially the attentional blink (Marois et al., 2000), a phenomenon of attentional selection occurring at a capacity limited stage that constrains the ability to consciously perceive different targets (Duncan et al., 1994). Since patients were trained to use their prosthesis, they may increasingly have recruited the right IPC during the training, which may have been followed by increased cortical thickness after the training. Additionally, we found that the more pain reduction the patients had after the training the less cortical thickness increase was found in this region. This goes in line with our former study (Preißler et al., 2013a). There, we observed a positive association between the cortical volume of right IPC and prosthetic use only in patients with high PLP (Preißler et al., 2013a). The more patients used their artificial device, the more volume was detected in this area. This was interpreted as a higher amount of visual attentional selection due to the use of the artificial device. In the resent study, somatosensory feedback about grip force may reduce the amount of visual attention necessary to control the prosthesis. This reduction over the training period seems to be modulated by changes in PLP.

We showed a reduction of cortical thickness in the right DLPFC after training. This is in line with previous studies showing DLPFC activation in the presence of a mismatch between motor intention and sensory or visual feedback (Fink et al., 1999). Patients got SF based on grip force during the training, which should mirror their motor intention. In the course of the training, the mismatch between motor intention and sensory feedback should be fundamentally reduced, because patients now receive somatosensory information on grip force. This might indicate a lower recruitment of the DLPFC, which may be followed by a reduction of cortical thickness after the training.

#### Cortical changes caused by the PLP change controlling for training effects

##### Areas related to pain processing

We observed a positive relation between PLP change and an increase in cortical thickness in post-central cortex on the right hemisphere, i.e., ipsilateral to the amputation side, indicating that the larger the reduction of pain following training, the smaller the increase in cortical thickness. This result corresponds to results on stroke patients where CIMT of the affected arm is associated with a reduction of the cortical motor area ipsilateral to the affected arm (Liepert et al., 1998). Our training is based on principles of CIMT, i.e., patients were instructed to use the affected arm as frequently as possible and to refrain from using the healthy arm as consequently as possible. Besides this requirement, less PLP following training might also result from an increased use of the affected side/the prosthesis. This again should be followed by less compensatory behavior with the healthy side. It is likely that more reduction of PLP is accompanied by less increase in post-central cortex ipsilateral to the amputation.

In both hemispheres, we showed a positive association between a PLP change following training and a change in cortical thickness in the ACC. This region is part of the anterior midcingulate cortex (aMCC; Vogt et al., 2003). These results confirm our former study where we reported an increased gray matter volume following training in patients suffering from high PLP compared to patients with less PLP (Preißler et al., 2013b). This result points toward a specific role of the aMCC in pain processing. It has been shown, that the aMCC is associated with affect encoding of pain (Rainville et al., 1997). Furthermore, it has been demonstrated that it plays a role in anticipatory attention toward emotionally salient (e.g., painful) stimuli (Bentley et al., 2003). Besides this, the aMCC is also commonly activated after nociceptive stimulation (Peyron et al., 2000). Our observations do not correspond to those reported by May (2011b). The author observed a reduction of gray matter in areas involved in experience and anticipation of pain as the ACC (May, 2011b). However, we showed a positive association between PLP and gray matter in the ACC in a former study (Preißler et al., 2013b) and hypothesized that this might reflect the permanent unpleasant pain experience. The positive effect of our treatment on PLP might have adjusted the cortical thickness of the ACC.

We found a positive relation between PLP change and a smaller increase in cortical thickness in the DLPFC bilaterally. This observations correspond with findings of Lorenz et al. (2003). They investigated 14 right-handed male subjects using PET during a painful stimulation to examine whether the frontal lobe is involved in top-down or bottom-up processing of noxious input. The authors concluded that the DLPFC is associated with an active control on pain perception. A person experiencing less PLP after the training may need less active control of the pain, which seems to go in line with less recruitment of the DLPFC followed by less cortical thickness in this region.

##### Areas related to body owner ship and visual control

We found a negative association between the PLP change during the training and the reduction of cortical thickness in areas of the visual pathways bilaterally; the higher the PLP reduction, the lower the decrease in gray matter in these areas after the training. On the left hemisphere, there is a reduced thickness in the middle temporal gyrus whereas on the right hemisphere we found a similar reduction in the occipital gyrus. In a former study, we showed an increased gray matter in the visual stream of patients with low PLP in comparison to patients with strong PLP (Preißler et al., 2013b). These findings are in line with the results of the actual study. A reduced PLP after training is associated with less reduction of these areas. Apparently, PLP intensity differs in relation to the amount of visual control.

We found a positive association between the reduction of PLP following training and the reduction of cortical thickness in the posterior cingulate cortex (PCC; right hemisphere). The PCC is discussed as an important structure for visuospatial orientation, topokinesis, and navigating the body in space (Vogt, 2005). Simultaneously, the PCC reflects an area of self-reflection and was shown to be inactive in response to nociceptive stimulation (Vogt et al., 1996). Inactivation of this region could be one mechanism to reduce the overall perception of noxious stimulation and reduce suffering (Vogt and Laureys, 2005). Therefore, an increase of thickness may reflect the effort for less suffering.

#### Joint reflection of the results

Concerning cortical thickness, we found effects of the training, i.e., reductions in cortical thickness in the left middle temporal cortex, left aMCC, right occipital cortex, right superior frontal cortex, and right DLPFC, and a gain in cortical thickness in the right IPC. These effects survive when controlling for PLP change probably induced by the training.

Furthermore, there are separate effects of PLP change on cortical thickness. These effects did not influence, suppress, or even enhance the effects of the training on morphometry. Apparently, the association between cortical thickness and areas reported for pain processing is only associated with PLP change during the training and does contribute to training effects. There is also one region in the middle temporal cortex, belonging to the visual stream, where both PLP changes and training are superimposed. In addition, the change in cortical thickness during the training is enhanced by effects of pain change in SI of the right hemisphere. In contrast, the associations between PLP change and cortical thickness changes, on the one hand, and between training effects and cortical thickness changes, on the other hand, in the right IPC and in the right DLPFC are opposite.

#### Limitations and future directions

As we studied a rare patient group, a limitation of our study is the small sample size, thus, it is possible that many effects observed in these patient reflect specific morphological changes of amputation. Therefore, future studies should focus more on the intra- and inter-individual differences of morphometry. This has already been proposed for functional studies on pain (e.g., Kucyi and Davis, 2015), but should also be used for structural research.

Due to technical development in the course of this research project, the patients were measured on two different MRI scanners with different field strengths. All image data for each single patient were conducted on the same scanner, so that all changes are related to differences on the same scanner. Additionally, using the longitudinal approach of freesurfer the scanner specific effects should be averaged out. Nevertheless, we cannot fully exclude that the different scanners may have influenced our results.

Furthermore, our training period was rather short. We believe, and so did many patients treated, that longer training periods and/or a more intensive integration of SF into the training and its transfer to daily life would increase the effects on both, the behavioral und morphological levels.

Additionally, we had only one female participant. Therefore, we cannot conclude that our results are transferable to female patients with upper limb amputation.

Moreover, age may have an influence on the training success and on morphological changes due to training processes. We tried to discuss only data, where age seems to have no influence. However, a detailed investigation of age effects is beyond the scope of the current study.

## Author contributions

All authors follow the criteria of authorship. The authors provide substantial contributions to the conception and design of the work (SP, DT, GH, WM, and TW), and the acquisition (SP, DT, and CD), analysis (SP and DT), and interpretation of data (SP, DT and TW). All authors worked together on the manuscript. They provided important intellectual content to draft the work (SP and DT) and to revise it critically (SP, DT, CD, GH, WM, and TW). All authors approved the final version to be published (SP, DT, CD, GH, WM, and TW) and agreed to be accountable for all aspects of the work in ensuring that questions related to the accuracy or integrity of any part of the work are appropriately investigated and resolved.

### Conflict of interest statement

The authors declare that the research was conducted in the absence of any commercial or financial relationships that could be construed as a potential conflict of interest.

## Supplementary material

Figure S1 Cortical thickness differences due to the training with *N* = 9 (male). Inflated presentation of a standardized brain. Areas shown in blue are regions with decreased cortical thickness after the training. Only areas with a vertex-wise threshold of *p* > 0.05 are shown. 1, supramedial; 2, middle temporal; 3, anterior cingulate, caudal part; 4, post-central; 5, inferior parietal; 6, occipital; 7, superior frontal cortex.

Figure S2 Cortical thickness differences due to the training adjusted by the covariate age (β_0_). Inflated presentation of a standardized brain. Areas shown in blue are regions with decrease in thickness after the training. Red areas mark an increase in thickness. Only areas with a vertex-wise threshold of *p* > 0.05 are shown. 1, middle temporal; 2, occipital; 3, anterior cingulate, caudal part; 4, superior frontal cortex; 5, post-central.

Table S1 Main effect of training (*N* = 9 men).

Table S2 Main effect of training adjusted for age.
