# Supplementary figures and images for: Preliminary Evidence for Training-Induced Changes of Morphology and Phantom Limb Pain
Source: Front Hum Neurosci. 2017 Jun 20;11:319. doi: 10.3389/fnhum.2017.00319 (PMC5476738; doi:10.3389/fnhum.2017.00319)

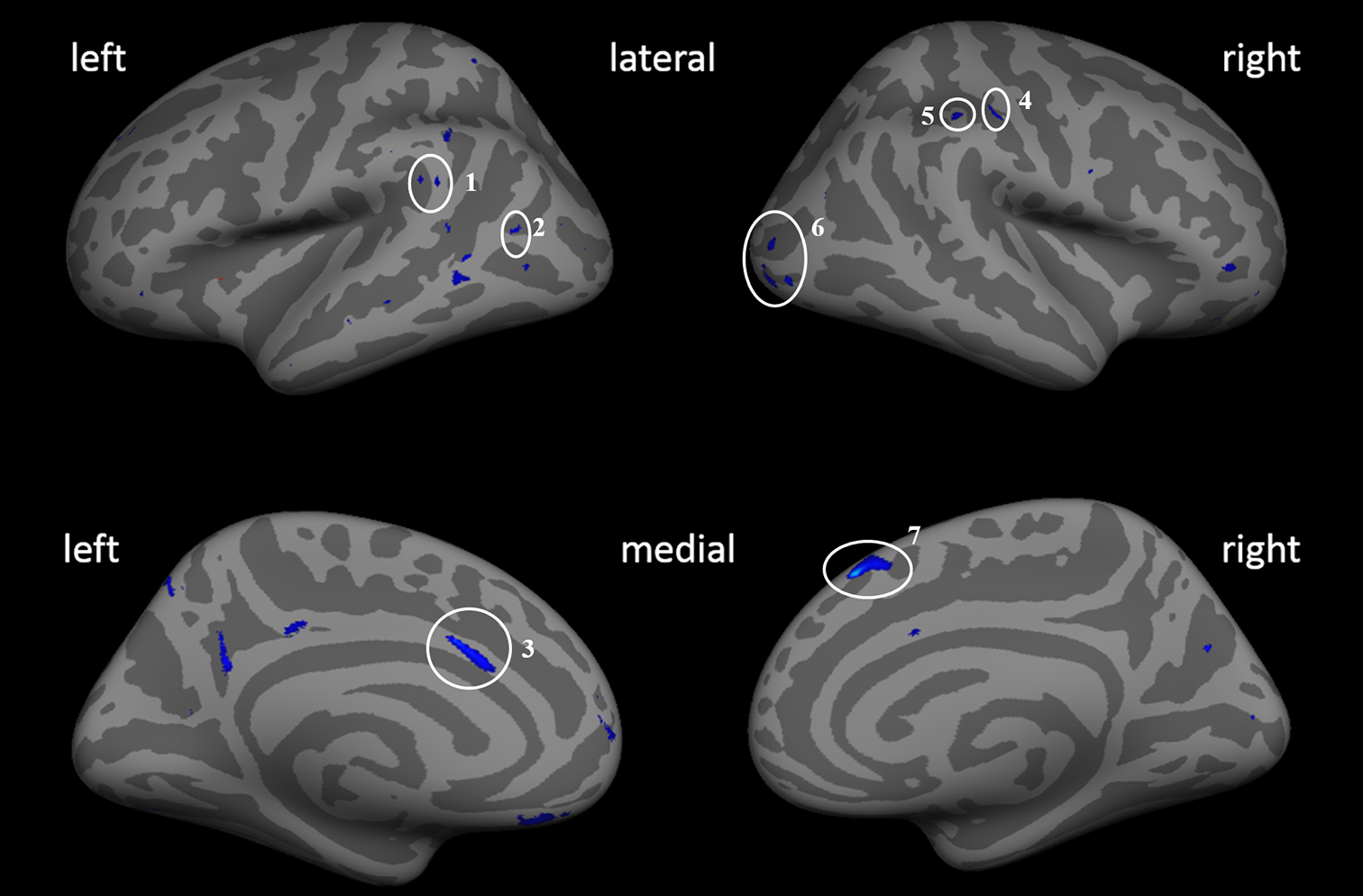

Supplement: Figure S1 — Cortical thickness differences due to the training with N = 9 (male). Inflated presentation of a standardized brain. Areas shown in blue are regions with decreased cortical thickness after the training. Only areas with a vertex-wise threshold of p > 0.05 are shown. 1, supramedial; 2, middle temporal; 3, anterior cingulate, caudal part; 4, post-central; 5, inferior parietal; 6, occipital; 7, superior frontal cortex. [file Image1.TIF]

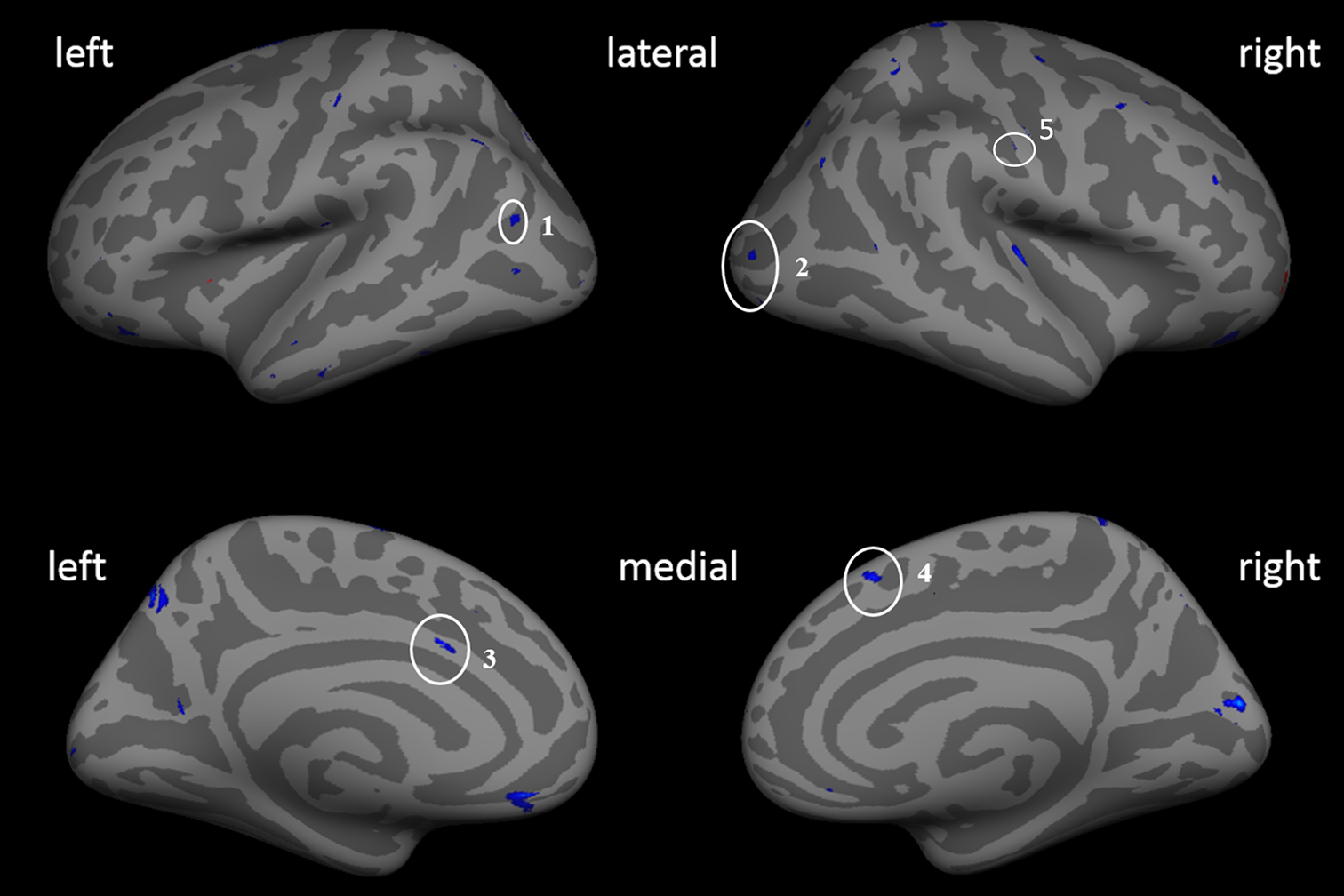

Supplement: Figure S2 — Cortical thickness differences due to the training adjusted by the covariate age (β0). Inflated presentation of a standardized brain. Areas shown in blue are regions with decrease in thickness after the training. Red areas mark an increase in thickness. Only areas with a vertex-wise threshold of p > 0.05 are shown. 1, middle temporal; 2, occipital; 3, anterior cingulate, caudal part; 4, superior frontal cortex; 5, post-central. [file Image2.TIF]
