# Supplementary material for: Preliminary Evidence for Training-Induced Changes of Morphology and Phantom Limb Pain
Source: Front Hum Neurosci. 2017 Jun 20;11:319. doi: 10.3389/fnhum.2017.00319 (PMC5476738; doi:10.3389/fnhum.2017.00319)
Supplement: Table S1 — Main effect of training (N = 9 men). [file Table1.DOC]

**Table S1. Main effect of training (N=9 men)**

| **Region** | **Size (mm^2)** | **maximal activation**  **(z-value)** | **Talairach coordinates** | | |
| --- | --- | --- | --- | --- | --- |
|  |  |  | **x** | **y** | **z** |
| Left hemisphere |  |  |  |  |  |
| Anterior cingulate cortex | 47.24 | -3.777 | -6.1 | 14.5 | 28.7 |
|  | 30.63 | -3.225 | -6.2 | 32.0 | -21.8 |
| Posterior cingulate cortex (BA 31) | 15.95 | -3.088 | -9.2 | -49.4 | 25.5 |
|  | 5.32 | -2.570 | -5.0 | -29.0 | 36.9 |
| Parietal cortex, precuneus | 26.64 | -3.175 | -7.2 | -66.3 | 49.6 |
|  | 4.85 | -2.865 | -12.8 | -50.7 | 29.7 |
| Temporal cortex (BA 22) | 7.38 | -2.862 | -55.6 | -53.5 | 6.2 |
| Right hemisphere |  |  |  |  |  |
| Superior frontal cortex (BA 8) | 84.36 | -4.756 | 7.1 | 30.1 | 46.5 |
| Postcentral gyrus | 8.43 | -2.868 | 46.9 | -20.0 | 38.0 |
| Inferior parietal lobule (BA 40) | 19.65 | -3.143 | 44.1 | -32.2 | 40.3 |
| Occipital cortex | 26.33 | -3.290 | 30.9 | -90.1 | -3.9 |
|  | 14.07 | -3.051 | 37.5 | -83.1 | -1.8 |
