# Supplementary material for: Preliminary Evidence for Training-Induced Changes of Morphology and Phantom Limb Pain
Source: Front Hum Neurosci. 2017 Jun 20;11:319. doi: 10.3389/fnhum.2017.00319 (PMC5476738; doi:10.3389/fnhum.2017.00319)
Supplement: Table S2 — Main effect of training adjusted for age. [file Table2.DOC]

**Table S2. Main effect of training adjusted for age**

| **Region** | **Size (mm^2)** | **maximal activation**  **(z-value)** | **Talairach coordinates** | | |
| --- | --- | --- | --- | --- | --- |
|  |  |  | **x** | **y** | **z** |
| Left hemisphere |  |  |  |  |  |
| Parietal cortex, precuneus | 60.61 | -3.694 | -8.0 | -69.1 | 44.8 |
|  | 26.13 | -3.150 | -6.2 | -64.8 | 44.8 |
| Dorsolateral prefrontal cortex | 28.71 | -3.639 | -8.6 | 36.2 | -14.3 |
| Anterior cingulate cortex | 7.97 | -3.382 | -6.2 | 16.4 | 27.4 |
|  | 12.07 | -2.677 | -5.3 | 36.7 | 18.7 |
| Superior frontal gyrus (BA 6) | 45.43 | -3.156 | -15.3 | -3.8 | 62.9 |
| Posterior cingulate cortex | 9.29 | -3.138 | -16.2 | -55.4 | 16.6 |
| Occipital cortex (BA 17) | 10.21 | -2.817 | -13.7 | -93.1 | 6.5 |
| Postcentral gyrus (BA 3) | 5.32 | -2.733 | -46.3 | -15.7 | 50.2 |
| Right hemisphere |  |  |  |  |  |
| Occipital cortex | 61.17 | -4.335 | 7.0 | -80.3 | 16.5 |
|  | 9.88 | -3.022 | 28.3 | -82.5 | 6.7 |
| Inferior parietal cortex | 7.71 | -3.512 | 40.5 | -68.0 | 35.6 |
| Temporal cortex | 25.50 | -3.476 | 40.2 | -24.2 | 6.6 |
| Precentral gyrus (BA 4) | 34.75 | -3.310 | 15.7 | -30.4 | 68.0 |
|  |  |  |  |  |  |
| Dorsolateral prefrontal cortex | 7.91 | -3.081 | 39.3 | 35.2 | 23.7 |
|  | 16.95 | -3.230 | 7.3 | 20.6 | 47.5 |
| Frontal cortex | 8.09 | -2.757 | 39.9 | 13.2 | 46.9 |
|  | 51.57 | -2.750 | 24.2 | 38.4 | -11.2 |
| Postcentral gyrus (BA 5) | 11.16 | -2.691 | 12.5 | -41.9 | 68.7 |
| Postcentral gyrus | 1.04 | -2.346 | 50.0 | -12.8 | 35.0 |
